# Selection of an Optimal Metabolic Model for Accurately Predicting the Hepatic Clearance of Albumin-Binding-Sensitive Drugs

**DOI:** 10.3390/ph18070991

**Published:** 2025-07-01

**Authors:** Ren-Jong Liang, Shu-Hao Hsu, Hsueh-Tien Chen, Wan-Han Chen, Han-Yu Fu, Hsin-Ying Chen, Hong-Jaan Wang, Sung-Ling Tang

**Affiliations:** 1 Clinical Pharmacy Department, Tri-Service General Hospital Keelung Branch, Keelung City 202006, Taiwan; 810010009@mail.ndmctsgh.edu.tw; 2 Graduate Institute of Medical Sciences, National Defense Medical Center, Taipei 114201, Taiwan; 3 School of Pharmacy, National Defense Medical Center, Taipei 114201, Taiwan; hsu.shuhao@mail.ndmctsgh.edu.tw (S.-H.H.); ccjh097429@gmail.com (H.-T.C.);; 4 Department of Pharmacy Practice, Tri-Service General Hospital, Taipei 114202, Taiwan; 5 Graduate Institute of Life Science, National Defense Medical Center, Taipei 114201, Taiwan

**Keywords:** hepatic clearance, in vitro-to-in vivo extrapolation (IVIVE), isolated perfused rat liver (IPRL), well-stirred model (WSM), modified well-stirred model (MWSM), protein binding

## Abstract

**Background/Objectives**: Hepatic clearance is important in determining clinical drug administration strategies. Achieving accurate hepatic clearance predictions through in vitro-to-in vivo extrapolation (IVIVE) relies on appropriate model selection, which is a critical step. Although numerous models have been developed to estimate drug dosage, some may fail to predict liver drug clearance owing to inappropriate hepatic clearance models during IVIVE. To address this limitation, an in silico-based model selection approach for optimizing hepatic clearance predictions was introduced in a previous study. The current study extends this strategy by verifying the accuracy of the selected models using ex situ experimental data, particularly for drugs whose model choices are influenced by protein binding. **Methods**: Commonly prescribed drugs were classified according to their hepatic extraction ratios and protein-binding properties. Building on previous studies that employed multinomial logistic regression analysis for model selection, a three-phase classification method was implemented to identify five representative drugs: diazepam, diclofenac, rosuvastatin, fluoxetine, and tolbutamide. Subsequently, an isolated perfused rat liver (IPRL) system was used to evaluate the accuracy of the in silico method. **Results**: As the unbound fraction increased for diazepam and diclofenac, the most suitable predictive model shifted from the initially preferred well-stirred model (WSM) to the modified well-stirred model (MWSM). For rosuvastatin, the MWSM provided a more accurate prediction. These three capacity-limited, binding-sensitive drugs conformed to the outcomes predicted by the multinomial logistic regression analysis. Fluoxetine was best described by the WSM, which is consistent with its flow-limited classification. For tolbutamide, a representative capacity-limited, binding-insensitive drug, no significant differences were observed among the various models. **Conclusions**: These findings demonstrate the accuracy of an in silico-based model selection approach for predicting liver metabolism and highlight its potential for guiding dosage adjustments. Furthermore, the IPRL system serves as a practical tool for validating the accuracy of the results derived from this approach.

## 1. Introduction

The liver is the primary organ involved in drug metabolism in humans. Since 1972, hepatic clearance (*CL_H_*) has been widely studied and increasingly used in clinical practice [1]. One method for accurate *CL_H_* predictions is in vitro-to-in vivo extrapolation (IVIVE), which eliminates the need for extensive animal experiments [2]. However, the accuracy of IVIVE relies on appropriate model selection, which is a critical step in the IVIVE process. Numerous models have been developed to predict hepatic drug metabolism [3,4,5,6,7,8,9,10,11,12,13]. Among these models, the well-stirred [7], parallel-tube [14], and dispersion [15] models (WSM, PTM, and DM, respectively) are commonly used to estimate *CL_H_*. However, these models sometimes fail to evaluate drug metabolism accurately, leading to suboptimal therapeutic outcomes or toxicity. Previous investigations have shown that the traditional WSM (Equation (1), *CL_H_* represents the hepatic clearance, *Q_H_* is the hepatic blood flow, *f_u_* is the unbound fraction in plasma, and *CL_int_* is the intrinsic clearance of the drug) may not reliably predict the metabolic profiles of albumin-binding-sensitive drugs. In 2021, a refined version of the WSM, referred to as the modified WSM (MWSM; Equation (2)), was introduced to provide a more precise and comprehensive assessment of drug metabolism under defined conditions [6].

(1) $${CL}_{H}=\frac{Q_{H}\times f_{u}\;\cdot \;{CL}_{int}}{Q_{H}+f_{u}\;\cdot \;{CL}_{int}}\;$$

(2) $${CL}_{H}=f_{u}\;\cdot \;{CL}_{int}\;$$

Building on the principles of IVIVE, we previously established a categorized approach using in silico analysis to guide the selection of drug clearance models. This approach was developed based on 16 detailed physicochemical and pharmacokinetic parameters from 223 compounds and was derived through multinomial logistic regression analysis [3]. In the present study, to validate the predictive accuracy of this approach, we used it to determine the optimal *CL_H_* model for three representative drugs: diazepam, diclofenac, and rosuvastatin. Each drug was classified according to one of the following three outcomes: W (better prediction with WSM), M (better prediction with MWSM), or S (equally accurate prediction with both models). The probabilities for each outcome were calculated, and the category with the highest probability was further verified by ex situ results obtained from liver perfusion experiments. This framework provides a robust data-driven methodology for determining the most suitable hepatic model based on drug-specific profiles, offering a systematic and optimized approach for enhancing the accuracy of IVIVE predictions.

**Figure 1:**
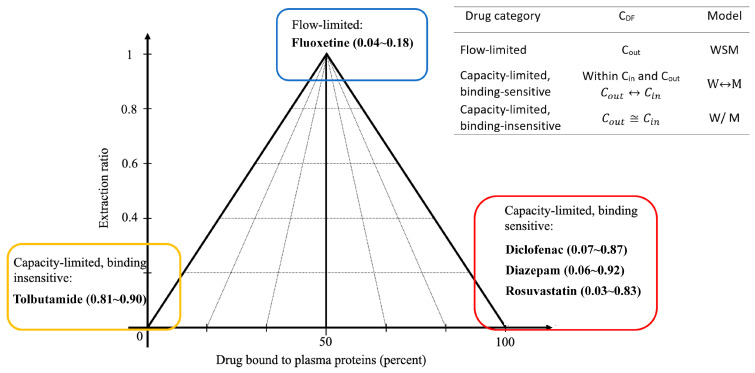
Three-phase classification is based on different degrees of liver extraction ratio and plasma protein binding. The numbers in parentheses represent the range of liver bioavailability, as the protein concentration is adjusted. WSM or W: well-stirred model; M: modified well-stirred model; *C_DF_*: driving force concentrations; *C_in_*: drug concentration entering liver; *C_out_*: drug concentration exiting liver.

In this study, the aforementioned strategy was experimentally validated. First, a widely used three-phase classification scheme was employed to identify the representative drugs from each category [16]. According to established pharmacokinetic concepts [8], flow-limited drugs (Figure 1, upper apex of the triangle) generally align with the “WSM recommended” category, whereas capacity-limited, binding-insensitive drugs (Figure 1, lower left of the triangle) tend to show “no significant difference across models.” However, capacity-limited binding-sensitive drugs (Figure 1, lower right) represent more complex situations. For instance, a previous report [6] indicated that diazepam cannot always be described using a single model under varying protein concentrations. Therefore, a previously established in silico-based method was employed in the study to predict the optimal *CL_H_* model for such drugs. These compounds were then subjected to isolated perfused rat liver (IPRL) experiments with different protein concentrations to measure the *CL_H_*. The resulting data verified the accuracy of the model selection by in silico analysis and provided a basis for further discussion of the drug–protein-binding effect.

Five representative drugs were selected for this study. Fluoxetine is a flow-limited drug [17]; tolbutamide is a capacity-limited, binding-insensitive drug [18]; and diazepam, diclofenac, and rosuvastatin are capacity-limited, binding-sensitive drugs [19,20,21,22]. First, an in silico multinomial logistic regression analysis was conducted to predict the most suitable *CL_H_* model for binding-sensitive drugs. The “percent better prediction” (%BP) value was then used to indicate the extent to which a particular *CL_H_* model was favored. Subsequently, an IPRL experiment was performed for each drug at different protein concentrations to measure actual *CL_H_*, and the resulting data were subjected to statistical analysis to determine which model was more appropriate, thereby elucidating the influence of proteins on hepatic drug metabolism. These experimental findings are intended to validate the feasibility of using an in silico model selection approach for IVIVE of drugs with diverse physicochemical and pharmacokinetic properties. This method reduces the time required to identify an appropriate *CL_H_* model and provides valuable guidance for clinical dose adjustment. Compared with our previous works that primarily focused on theoretical modeling [3,6], the current study extends the methodology by validating the logistic regression-based model selection across multiple drug classes. This integration of in silico prediction with ex situ IPRL results not only reinforces the mechanistic basis but also broadens the model applicability under varied physiological conditions.

## 2. Results

### 2.1. Logistic Regression Modeling

Based on logistic regression analysis for selecting hepatic models, the category with the highest probability, reflected by the BP%, was identified as the optimal hepatic model (W, M, or S). None of the drugs tested exhibited the highest probability in category S. As presented in Table 1, the WSM exhibited significantly higher BP% values than the MWSM for diazepam and diclofenac (92.92% vs. 0.04% and 55.26% vs. 13.92%, respectively), indicating the superior predictive performance of the WSM for these compounds. In contrast, MWSM outperformed WSM in terms of the BP% for rosuvastatin (48.59% vs. 19.95), demonstrating MWSM’s greater suitability for this drug. The other two test drugs, fluoxetine and tolbutamide, were not evaluated by logistic regression analysis. This is because fluoxetine, classified as a flow-limited drug, has metabolic characteristics inherently captured by WSM. Conversely, tolbutamide, which is categorized as a capacity-limited binding-insensitive drug, exhibited comparable predictive performance with both WSM and MWSM. In other words, both models predicted tolbutamide metabolism with similar accuracy. These results are consistent with observations from the IPRL, which are detailed in the following section.

### 2.2. Equilibrium Dialysis

**Figure 2:**
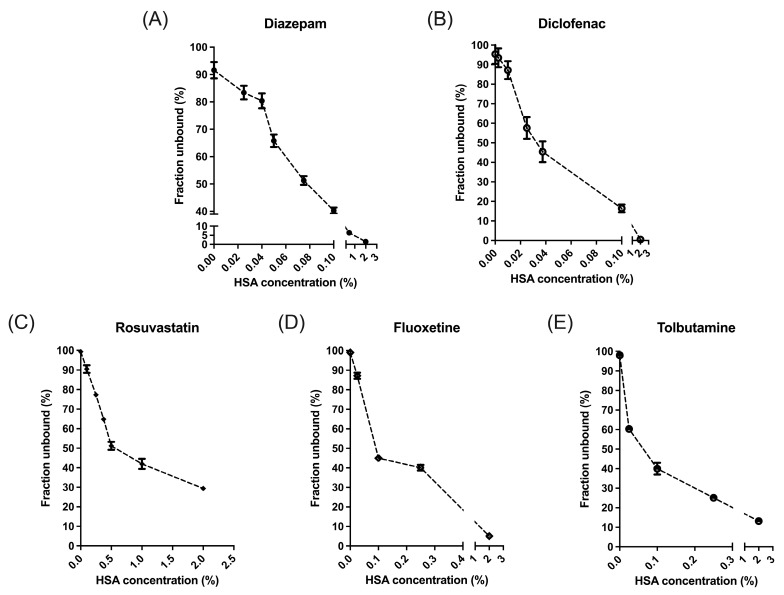
Different protein concentrations correspond to specific fraction unbound values using the rapid equilibrium dialysis method. (**A**) Data obtained from Hsu et al., 2021 [6]. (**B**–**E**) Data obtained in this study. The parameter *f_u_* was the most sensitive to changes in HSA concentration. HSA: human serum albumin; *f_u_*: fraction of unbound drug in blood.

The fraction unbound (*f_u_*) of each assessed drug at various HSA concentrations was determined by RED. For diclofenac, the *f_u_* values at 0%, 0.0025%, 0.01%, 0.025%, 0.1%, and 2% albumin were 1.000, 0.931, 0.872, 0.576, 0.164, and 0.006, respectively (Figure 2B), with the most pronounced changes occurring between 0.01% and 0.025%. Similarly, the *f_u_* values of rosuvastatin were 0.99, 0.90, 0.77, 0.65, 0.51, 0.42, and 0.29 (Figure 2C); those of fluoxetine were 0.99, 0.87, 0.45, 0.40, and 0.05 (Figure 2D); and those of tolbutamide were 0.98, 0.60, 0.40, 0.25, and 0.13 (Figure 2E), respectively.

### 2.3. Drug Metabolism in IPRL

**Figure 3:**
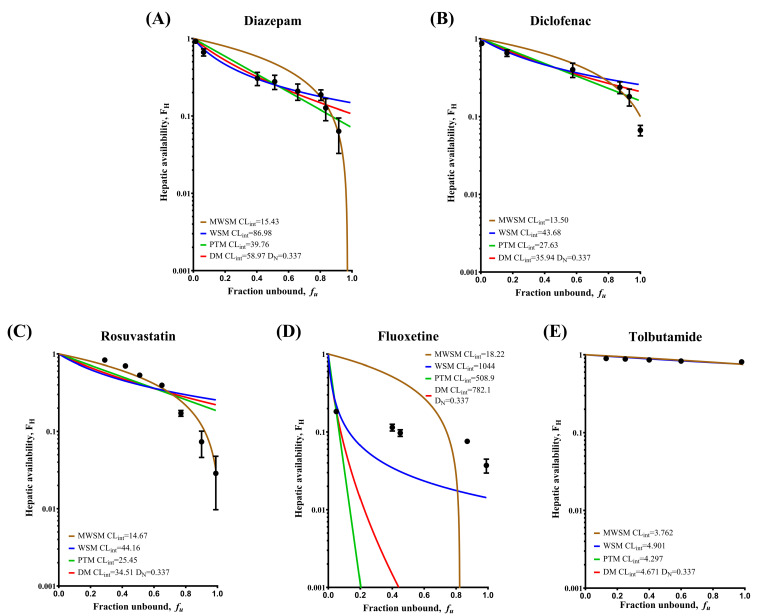
Differences in the hepatic availability (*F_H_*) of different drugs at specific unbound fractions. According to an average value of *f_u_* at the cutoff point, the simulated curves indicated in different colors were obtained using MWSM (brown), WSM (blue), PTM (green), and DM (red) models. (**A**) Data were obtained from a previous study [6]. The transformation point of diazepam is set at *f_u_* = 0.804. MWSM is simulated using the *f_u_* range between 0.804 and 0.916. WSM, PTM, and DM are simulated using the *f_u_* range between 0.015 and 0.804. (**B**) The transformation point of diclofenac is set between *f_u_* = 0.576 and 0.872. MWSM is simulated using the *f_u_* range between 0.872 and 0.954. WSM, PTM, and DM are simulated using the *f_u_* range between 0.006 and 0.576. (**C**–**E**) There are no transformation points for any of the assessed drugs. For each drug, all models were simulated using the entire *f_u_* range. All points in the figure represent average IPRL values for specific protein binding with a different cutoff point. IPRL: isolated perfused rat liver; HSA: human serum albumin; *f_u_*: fraction of unbound drug in blood; *CL_int_*: intrinsic clearance; WSM: well-stirred model; MWSM: modified well-stirred model; PTM: parallel-tube model; DM: dispersion model.

In this part of the analysis, each drug exhibited a distinct *f_u_* at different protein concentrations. Under this condition, hepatic availability (*F_H_*) served as a clear and practical indicator of *CL_H_*. A higher *F_H_* (ranging from 0 to 1) reflected a lower degree of hepatic metabolism and clearance. Based on the principles of the WSM, Equations (3) and (4) were derived by applying Equations (1) and (2), in conjunction with the concept that *CL_H_* equals the liver blood flow multiplied by the extraction ratio. Subsequently, data fitting was performed using these formulas. Specifically, after listing the *F_H_* equations for each *CL_H_* model (Equations (3) and (4)) and incorporating the liver blood flow rate from the IPRL experiment (Q_H_ = 15 mL/min), the data points obtained from the IPRL were fitted, as shown in Figure 3, to yield the optimal intrinsic clearance (*CL_int_*) for each model.

(3) $$F_{H}=\frac{Q_{H}}{{CL}_{int,WSM}\cdot f_{u}+Q_{H}}$$

(4) $$F_{H}=1-\frac{{CL}_{int,\;MWSM}}{Q_{H}}\cdot f_{u}$$

Table 2 summarizes how the *F_H_* for the five drugs varied at different protein (HSA) concentrations in the IPRL experiments. In addition to the WSM and MWSM discussed previously, commonly used PTM and DM were included for comparison. By substituting the *f_u_* values from Figure 2 in place of the HSA concentrations reported in Table 2, it becomes more intuitive to determine which model more accurately characterizes the *CL_H_* behavior of each drug, as illustrated in Figure 3. The details follow those described in our previous report [6].

As shown in Table 2, higher protein concentrations led to an increased F_H_, reflecting a decrease in the fraction of drug cleared by the liver. This outcome aligns with the logic that only the free drug is metabolized. Figure 3 further illustrates that when the *f_u_* of diazepam and diclofenac (Figure 3A,B) approaches 1, the overall F_H_ trends shift from closely resembling the WSM (blue line in Figure 3) to more closely matching the MWSM (brown line in Figure 3). Rosuvastatin (Figure 3C) consistently followed the MWSM pattern, highlighting the ability of MWSM to address the limitations of WSM for particular drugs. Fluoxetine (Figure 3D) appeared to display *F_H_* profiles in which WSM was still the best model for effective capture. Based on the obtained data, fluoxetine was considered to follow the WSM pattern, consistent with the expectation that flow-limited drugs would still be best described by the WSM for *CL_H_* calculations [8]. Tolbutamide (Figure 3E), which was relatively insensitive to changes in protein concentration, showed similar fits under all four models, aligning well with the IPRL experimental results shown in Table 2. Thus, each model demonstrated an equivalent capacity to describe the hepatic metabolic profile.

Different albumin concentration sequences were designed for each drug because the curve could be split into two intervals corresponding to the well-stirred model and the modified well-stirred model. This approach allowed for a more precise observation of the specific model by switching ranges for each drug.

### 2.4. Fitting the Model-Simulated Curves

By calculating the sum of the squares between the predicted and observed *F_H_* values shown in Figure 3, it was possible to quantitatively determine which *CL_H_* model best suited each drug (Table 3). Diazepam and diclofenac exhibited clear preferences for MWSM and WSM, respectively, at various HSA concentrations. Rosuvastatin consistently aligned with the MWSM predictions throughout the experiment. As anticipated, fluoxetine aligned more closely with the WSM, as evidenced by its lowest sum of squares value among all the tested models, indicating that the WSM remained the most suitable. Tolbutamide, the representative binding-insensitive, capacity-limited drug, showed sum of squares values below 0.1 for all models. This outcome indicated that all models could accurately predict the *CL_H_* of tolbutamide, which is associated with a poor degree of liver metabolism.

## 3. Discussion

**Figure 4:**
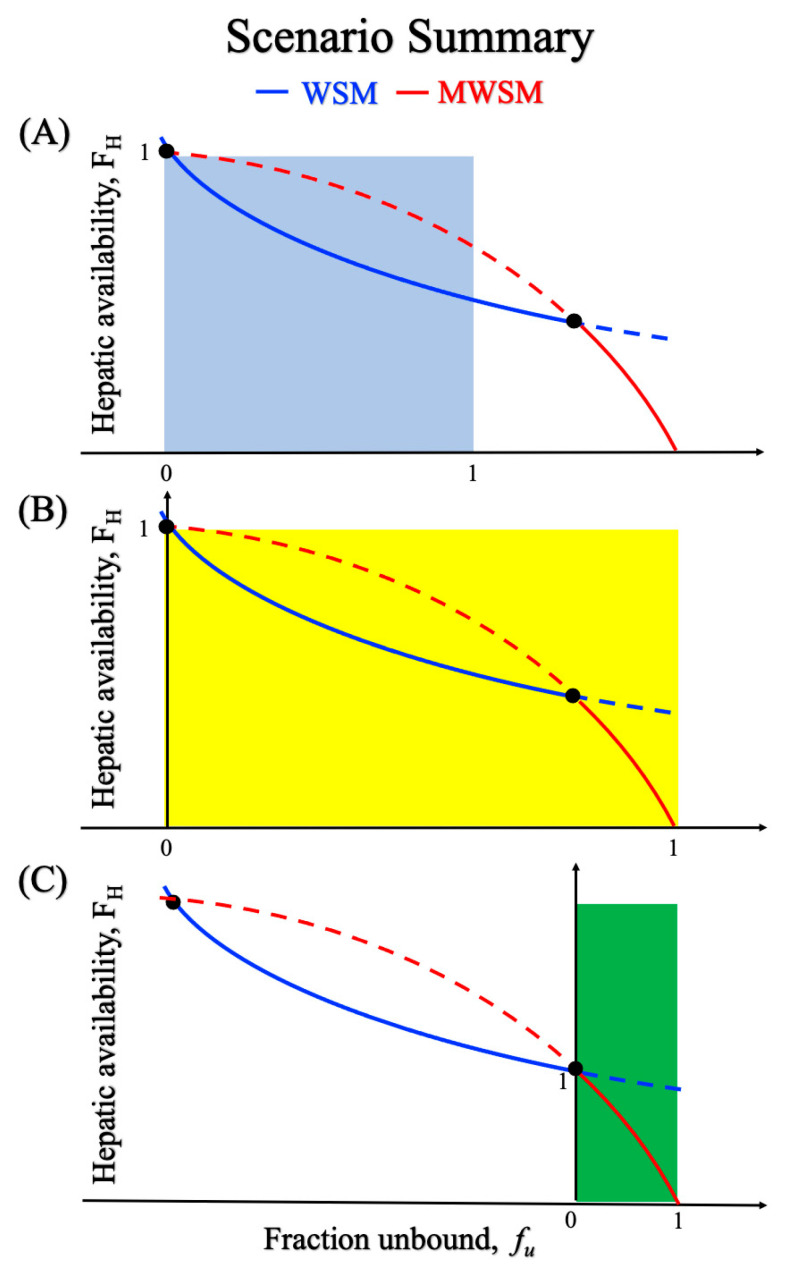
Four scenarios related to the classification of capacity-limited, binding-sensitive drugs. (**A**) The metabolism of the drugs in this scenario will always follow WSM. (**B**) When *f_u_* varies between 0 and 1, two intersection points will appear, indicating that as *f_u_* increases, the drug metabolism pattern will shift from WSM to MWSM. (**C**) In this scenario, drug metabolism consistently follows MWSM. Brown line: modified well-stirred model (MWSM); blue line: well-stirred model (WSM); black intersection point: the transition point of the metabolism model.

As shown in Figure 3 and Table 3, diazepam and diclofenac, both binding-sensitive capacity-limited drugs, exhibited a marked shift in model predictability as the protein concentration varied. This phenomenon can be explained by Equations (3) and (4), as shown in Figure 4. The underlying concept for Figure 4 is rooted in the free-drug hypothesis: If *f_u_* is zero, no drug is available for metabolism. Thus, both the WSM and MWSM curves begin from the point *F_H_*, *f_u_* = (1, 0).

To elucidate this phenomenon, Figure 4 is divided into three scenarios. As shown in Figure 4A, the drug was metabolized efficiently by the liver, leading the *F_H_* to align with the WSM trend under all *f_u_* values. The flow-limited drug, fluoxetine, served as an example. Figure 4B illustrates the situation for diazepam and diclofenac, where increasing *f_u_* prompts a transition from WSM to MWSM. Figure 4C shows that rosuvastatin consistently followed the MWSM trend, indicating the necessity of using the MWSM in liver metabolism research. For a capacity-limited, binding-sensitive drug, determining whether its behavior aligns more closely with scenario B or C requires a multifaceted approach, including in silico methods, to enhance the accuracy of IVIVE in predicting hepatic metabolic profiles.

In contrast to the aforementioned drugs, fluoxetine is considered to be representative of the flow-limited group of drugs, all of which are highly metabolized regardless of their protein-binding levels. Our IPRL results revealed a sharp decline in bound fluoxetine at high albumin concentration intervals, and the MWSM could not predict such rapid reductions in *F_H_* at such high albumin concentrations (Figure 3D). Therefore, these drugs can only be described using WSM to depict their metabolic profiles.

Drugs in the binding-insensitive group undergo minimal metabolism in the liver (low extraction ratio, ER); in this regard, we selected tolbutamide to verify the in silico-based model selection method. However, the poor metabolism of the drugs in this group resulted in only minimal metabolic differences among the different models, with all models showing similar predictive abilities.

Before exploring the effects of protein binding on *CL_H_*, it is instructive to systematically review the underlying assumptions of currently used *CL_H_* models to clarify some often-overlooked conceptual details. The apparent differences among these models, including WSM, PTM, DM, and MWSM, stem from the conceptualization of drug distribution within the liver. This distinction manifests as different “driving force concentrations” (*C_DF_*) in the derivation of the equations of each model. Specifically, *CL_H_* is defined as the ratio of the elimination rate of a drug to its concentration during metabolism (*C_x_*), as shown in Equation (5):

(5) $$CL=\frac{dx/dt}{C_{x}}=\frac{v}{C_{x}}$$

Under liver conditions, two primary assumptions are made: (1) at steady state, the instantaneous change in the elimination rate of a drug is zero, and (2) the proportion of biliary excretion is negligible. Consequently, changes in drug concentration are predominantly governed by liver metabolism. Under these conditions, the numerator in Equation (5) can be expressed as Equation (6):

(6) $$v=\frac{dx}{dt}=Q\;\cdot \;C_{in}-Q\;\cdot \;C_{out}$$

From a microscopic perspective, drug metabolism in the liver proceeds when free drug molecules bind to liver enzymes and undergo metabolic reactions (as illustrated in Equations (7) and (8)):

(7) $${CL}_{int}=\frac{\frac{dx}{dt}}{C_{x}\;\cdot \;f_{u}}=\frac{Q\;\cdot \;C_{in}-Q\;\cdot \;C_{out}}{C_{x}\;\cdot \;f_{u}}$$

(8) $${CL}_{int}\;\cdot \;f_{u}\;\cdot C_{x}=Q\;\cdot \;C_{in}-Q\;\cdot \;C_{out}$$

In standard *CL_H_* derivations, concentration *C_x_* is assumed to equal *C_out_* [7]. Rearranging the terms provides the expression for *C_in_* (Equation (9)). Substituting this into Equation (10) yields the classic form of the WSM (Equation (1)).

(9) $$C_{in}=\frac{\;C_{out}\;\cdot \;\left(Q+{CL}_{int}\;\cdot \;f_{u}\right)}{Q}$$

(10) $${CL}_{H}=Q\;\cdot ER=Q\;\cdot \;\frac{C_{in}-C_{out}}{C_{in}}$$

In the other *CL_H_* models, *C_x_* in Equation (8) is substituted with what each model deems to be the “true” *C_DF_* during hepatic drug metabolism. Nevertheless, these three critical points warrant further examination.

1. When protein binding occurs, does the variation in the concentration of the free drug influence the accuracy of model predictions, and if so, in what way?
2. WSM designates *C_out_* as the *C_DF_*. What are the reasons behind this choice and what mechanisms could validate this assumption?
3. While Equation (10) has long been regarded as practical and intuitive from a macroscopic perspective, questions remain regarding its universal applicability.

Previous experimental findings for diazepam and diclofenac indicate that when *f_u_* approaches 1, the optimal predictive model shifts from WSM to MWSM. In its derivation, the only difference between the MWSM and WSM is that the former designates *C_in_*, rather than *C_out_*, as the *C_DF_*. This observation suggests that when a drug exists predominantly in its free form, the *C_DF_* more closely approximates *C_in_*. In reality, a drug experiences several physicochemical processes, including diffusion, partitioning, and transmembrane transport, before encountering liver enzymes, and its concentration upon reaching these enzymes is unlikely to be exactly *C_in_*. This scenario becomes especially relevant when protein binding is considered, as a certain fraction of the drug is initially unable to undergo metabolism (assuming that protein-facilitated uptake or dissociation is minimal). Consequently, for binding-sensitive drugs, the *C_DF_* is effectively reduced by protein binding and is therefore closer to *C_out_* (as assumed by the WSM). However, once *f_u_* increases sufficiently, the greater availability of the free drug increases the *C_DF_* closer to *C_in_* (as assumed by the MWSM). Hence, whenever the *C_DF_* is altered by the complexity of the metabolic environment, the choice of the *CL_H_* model depends on whether this concentration is closer to *C_in_* or *C_out_*. Much of our investigation of *CL_H_* models focused on these distinctions. IPRL experiments with binding-sensitive, capacity-limited drugs have provided empirical evidence confirming that these considerations have practical relevance beyond mere theoretical constructs.

Flow-limited drugs are also susceptible to protein-binding effects. Why do they not exhibit model shifts when *f_u_* changes? This situation arises because drugs with high ER are considered “flow-limited,” implying that for the specific drug, the metabolic capacity of the liver is so great that any incoming drug is immediately metabolized, leaving liver blood flow as the only limiting factor. Interestingly, this description inherently aligns with the fundamental assumption of the WSM, namely that once the drug enters the liver, it is instantaneously distributed and metabolized throughout the organ. In reality, whether a drug is readily metabolized in the liver depends on two factors: (1) how easily free drug molecules travel from the bloodstream to the metabolic enzymes and (2) the intrinsic capacity of those enzymes to metabolize the drug. A compound that perfectly conforms to the WSM predictions would exhibit both a very high distribution potential (related to its physicochemical properties) and an especially high enzymatic capacity (pharmacokinetic attribute). However, not every high-ER drug satisfies these two criteria. Whenever either condition is not met, WSM-based predictions can become inaccurate and potentially deviate from the observed values by more than twofold [23]. This consideration helps clarify why fluoxetine, although appearing to follow the WSM to a great extent, still does not perfectly match the WSM predictions [17,24].

Although Equation (10), adapted from chemical engineering principles, was introduced early in the development of the *CL_H_* theory and underlies most current *CL_H_* models, related discussions remain active in this area. For instance, Benet et al. re-examined Equation (10) and its assumptions from the perspective of the *C_DF_* [25] and further indicated that Equation (8) is flow independent, whereas Equation (10) is flow dependent, making it evident that the two are not connectable [26]. While the ER in Equation (10) can represent, on a macro scale, the proportion of drugs metabolized by the liver and is thus linked to the concept of *CL_H_*, it cannot be equated directly to Equation (8), which describes the microscopic behavior of drug molecules during metabolism. This misalignment in descriptive focus can introduce complications in the complex environment of the liver and is a possible reason for the widespread underestimation of *CL_H_* in IVIVE [27]. Currently, only drugs with minimal hepatic metabolism, such as tolbutamide, appear to be unaffected by this discrepancy, leading to consistent metabolic profiles across various models.

In summary, selecting the most suitable *CL_H_* model begins with a three-phase classification of drugs. As shown in Figure 1, for capacity-limited, binding-sensitive drugs, multinomial logistic regression is crucial for determining the appropriate model; however, variations in *f_u_* for such drugs may result in a shift in the predictive model between WSM and MWSM. In contrast, WSM consistently provides the most accurate predictions for flow-limited drugs, whereas both WSM and MWSM exhibit comparable predictive accuracies for capacity-limited, binding-insensitive drugs. In addition, to the best of our knowledge, few studies have addressed the impact of hypoalbuminemia on *CL_H_* model selection. Our findings emphasize that the prediction models of *CL_H_* should not remain fixed but should instead be adjusted in response to changes in physiological albumin concentrations. Under hypoalbuminemic conditions, certain clinical drugs may require distinct *CL_H_* models to determine the proper dose. Since hypoalbuminemia is frequently observed in clinical scenarios such as chronic liver disease, malnutrition, and severe inflammation [28,29,30,31,32], accurate model selection under abnormal albumin levels is critical to avoid dosing errors and ensure therapeutic efficacy.

## 4. Materials and Methods

### 4.1. Logistic Regression Modeling for Hepatic Model Selection

In accordance with a previous study, we utilized multinomial logistic regression to predict the optimal hepatic model, either WSM or MWSM, for the selected compounds [3]. A formula (Equation (11)) was developed from the data on 223 compounds, incorporating 16 detailed physicochemical and pharmacokinetic parameters. The dependent variable representing the most suitable hepatic model was classified into three categories: W (WSM provides better predictions), M (MWSM provides better predictions), and S (both models yield equally accurate predictions). The probability of each outcome was determined by using a multinomial logistic regression formula (Equation (11)). The sum of all probabilities was 1.

(11) $$P\left(Y=k|X\right)=\frac{\exp\left(\beta_{k0}+\beta_{k1}X_{1}+\beta_{k2}X_{2}+\ldots +\beta_{kp}X_{p}\right)}{1+\sum_{j=1}^{K-1}\exp\left(\beta_{j0}+\beta_{j1}X_{1}+\beta_{j2}X_{2}+\ldots +\beta_{jp}X_{p}\right)}$$

where P(Y = *k*|*X*) represents the probability of a compound being classified into a specific category. *X*_1_, *X*_2_, … *X*_p_ are independent variables, including physicochemical and pharmacokinetic properties such as intrinsic clearance (*CL_int_*), protein-binding characteristics, and distribution volume (*V_ss_*). *β_kj_* denotes the regression coefficients for category *k* and variable *X_j_*. *k* is the total number of categories (*k* = 3, corresponding to W, M, and S).

The probabilities for model classification were derived from compound-specific physicochemical and pharmacokinetic properties, which are critical for understanding drug–hepatic metabolic interactions. Our previous study used 16 independent variables for analysis based on their theoretical relevance to drug metabolic behavior in the liver. These variables included intrinsic clearance, protein-binding characteristics (e.g., unbound fraction), and distribution volume (Table 4).

Diazepam, diclofenac, and rosuvastatin, which are classified as capacity-limited (binding-sensitive) drugs, were selected to estimate their probabilities for hepatic metabolism model classification. These probabilities were expressed as BP%. For each compound, multinomial logistic regression was used to calculate the category probability, with the highest category probability designated as the optimal hepatic model (W, M, or S). Additionally, fluoxetine and tolbutamide, which are categorized as flow-limited and capacity-limited (binding-insensitive) drugs, respectively, were included in this study. We aimed to demonstrate that these two drugs could be distinctly classified into hepatic metabolism models based on their intrinsic characteristics, without the need for multinomial logistic regression analysis. All relevant statistical analyses related to multinomial logistic regression were performed using SPSS Statistics 28 (IBM Corporation, Armonk, NY, USA). Statistical significance was determined using two-tailed *p*-values, with values of less than 0.05 considered indicative of a significant effect.

### 4.2. Chemicals

Diclofenac, rosuvastatin, fluoxetine, tolbutamide, atorvastatin, naproxen, and diazepam were purchased from Thermo Fisher Scientific (Bellefonte, PA, USA), Pfizer (New York City, NY, USA), Acros Organics (Geel, Belgium), Sigma-Aldrich (St. Louis, MO, USA), CombiBlocks (San Diego, CA, USA), and Cerilliant (Round Rock, TX, USA). The perfusion buffer used for the IPRL experiments consisted of taurocholate, sodium bicarbonate, and commercial Krebs–Ringer powder obtained from Acros Organics (Merck, Taipei, Taiwan) and Sigma-Aldrich. Fatty acid-free human serum albumin (HSA) was obtained from SeraCare (Milford, MA, USA).

### 4.3. Rapid Equilibrium Dialysis

The rapid equilibrium dialysis (RED) apparatus and test inserts were purchased from Thermo Fisher Scientific (Rockford, IL, USA). The unbound fraction of each assessed drug was determined using RED. Briefly, a drug was diluted to 1 μg/mL in Krebs buffer containing different concentrations of HSA. Five hundred microliters of the assessed drug and each of the different HSA concentrations were added to the sample chamber of an insert and 750 μL of blank buffer was added to the adjacent chamber. The loaded inserts were then assembled on a Teflon-based plate. The plate was then sealed with a self-adhesive lid and agitated at 20 rpm and 37 ± 1 °C using an oscillating shaker (Genepure Rocking Shaker, OSR205-01) for 8 h.

### 4.4. Surgery and Liver Perfusion

Male Sprague–Dawley rats (350–450 g; BioLASCO Co., Ltd., Taipei, Taiwan) were housed at the Laboratory Animal Center of the National Defense Medical Center and provided free access to water and food with a 12 h light/dark cycle. All animal experiments were approved by the Institutional Animal Care and Use Committee of the National Defense Medical Center (IACUC-20-164, Taipei, Taiwan) on 7 May 2020. Anesthesia, surgical procedures, and perfusion were performed as previously described [6]. The surgery and liver perfusion were executed at the National Defense Medical Center from 2020 to 2024. Perfusates containing the assessed drugs and different concentrations of HSA were freshly prepared and maintained at 4 °C after mixing. All cycles lasted 20 min. For each cycle, samples were collected at 16, 17, 18, 19, and 20 min after incubation. At the end of each IPRL experiment, the rat liver was perfused with HSA-free Krebs buffer as a quality control measure to confirm enzymatic activity. In different perfusion cycles, the livers were perfused with buffer containing HSA in a sequence from low to high concentrations to minimize cross-contamination and other confounding factors. Bile elimination can be disregarded because it accounts for only 0.2% of total metabolism [7].

### 4.5. Sample Preparation and Analysis

A perfusate sample (400 μL) containing diclofenac was combined with 80 μL (1 μg/mL) of naproxen (internal standard, IS), 150 μL of 1% formic acid, and 2 mL of extraction solvent (ethyl acetate: *n*-hexane = 2:3). The mixture was vortexed for 5 min, and the resulting upper organic phase was collected, dried, reconstituted in 80% methanol, and used to inject rats for experiments. In addition, 100 μL of perfusate samples containing rosuvastatin, fluoxetine, and tolbutamide were combined with 20 μL (1 μg/mL) of IS (atorvastatin, diazepam, and diazepam) and 10 μL of 0.1% formic acid. The mixture was extracted using an Oasis HLB 1cc extraction cartridge.

For the separation of diclofenac, we used Thermos BDS Hypersil C18 columns (4.6 × 100 mm, 5 mm; Thermo Fisher Scientific, Waltham, MA, USA); for the separation of rosuvastatin, fluoxetine, and tolbutamide, we used Waters Symmetry C18 columns (2.1 × 100 mm, 1.7 μm; Waters Corporation, Milford, MA, USA).

The column separation of diclofenac, rosuvastatin, fluoxetine, and tolbutamide was achieved using isocratic elution with different mobile phase compositions tailored to each compound. Diclofenac was separated using a mobile phase of 2 mM ammonium acetate and 0.1% formic acid in water mixed with 2 mM ammonium acetate and 0.1% formic acid in methanol in a 1:9 ratio. For rosuvastatin, the mobile phase consisted of 0.1% formic acid in water and 0.1% formic acid in a mixture of 90% acetonitrile and 10% methanol in a 1:3 ratio. Fluoxetine was analyzed using a mobile phase of 0.1% formic acid in water and 0.1% formic acid in acetonitrile in a 3:7 ratio. The separation of tolbutamide was conducted using 0.1% formic acid in water and 0.1% formic acid in methanol in a 1:4 ratio.

For the analysis of diclofenac (296.1/250.0), naproxen (231.3/185.0), rosuvastatin (482.0/258.0), atorvastatin (560.3/441.1), fluoxetine (310.2/44.2), and diazepam (285.1/154.0), the samples were analyzed by the ionization interface of an Applied Biosystems-Sciex API 3000 series triple-quadrupole mass spectrometer (Foster City, CA, USA) with an Agilent Technologies 1200 series high-performance liquid chromatograph (Böeblingen, Germany). For that of tolbutamide (271.1/91.1) and diazepam (285.1/193.0), the samples were measured by a Shimadzu Nexera LC-40B X3 ultrahigh-performance liquid chromatograph (Böblingen, Germany) with a Biosystems-Sciex API 4000 series triple-quadrupole mass spectrometer (Foster City, CA, USA). The extraction recovery, calibration curves and retention time of each compound are shown in Table S1, Figures S1 and S2, respectively. All available experimental data were included in the analysis. The formula and experimental data were processed by Prism software v10.4.1 (GraphPad Software, Inc) to generate figures and calculate the degree of fit of different models.

## 5. Conclusions

This study demonstrates the importance of appropriate model selection for accurately predicting *CL_H_* using an IVIVE method. By conducting experiments using an IPRL system, we validated the utility of the in silico-based model selection method for distinguishing between the WSM and MWSM models for predicting *CL_H_* across diverse drug categories. These findings underscore the importance of model selection in optimizing *CL_H_* predictions and emphasize the value of tailoring *CL_H_* models to the unique characteristics of each drug, with significant implications for dosing adjustments and clinical decision-making, particularly in patients with varying albumin levels.

## Figures and Tables

**Table 1:** Multinomial logistic regression for the percent better prediction of the application of different drugs based on the well-stirred and modified well-stirred models.

Drug	Percent Better Prediction (BP%)		
	W	S	M
Diazepam	92.92	7.04	0.04
Diclofenac	55.26	30.82	13.92
Rosuvastatin	19.95	31.46	48.59

S: Similar predictive power of the two models; M: modified well-stirred model; W: well-stirred model.

**Table 2:** Relevant hepatic bioavailability (mean ± SD) of assessed drugs at different albumin concentrations (n number shown in parentheses).

	Concentration Sequences	1	2	3	4	5	6	7	8
Drug									
Diazepam ^a^		0.063 ± 0.031 (15)	0.127 ± 0.040 (15)	0.188 ± 0.029 (9)	0.208 ± 0.050 (15)	0.277 ± 0.058 (9)	0.305 ± 0.059 (15)	0.664 ± 0.070 (6)	0.920 ± 0.040 (6)
Diclofenac ^b^		0.067 ± 0.010 (6)	0.180 ± 0.044 (5)	0.237 ± 0.041 (6)	0.400 ± 0.084 (6)	0.654 ± 0.064 (6)	0.869 ± 0.039 (4)	-	-
Rosuvastatin ^c^		0.029 ± 0.019 (12)	0.073 ± 0.027 (6)	0.172 ± 0.015 (6)	0.395 ± 0.015 (6)	0.533 ± 0.037 (6)	0.700 ± 0.032 (6)	0.833 ± 0.039 (4)	-
Fluoxetine ^d^		0.037 ± 0.008 (7)	0.076 ± 0.005 (7)	0.097 ± 0.010 (7)	0.114 ± 0.011 (7)	0.183 ± 0.010 (6)	-	-	-
Tolbutamide ^d^		0.808 ± 0.030 (6)	0.828 ± 0.025 (6)	0.858 ± 0.015 (6)	0.880 ± 0.014 (6)	0.895 ± 0.014 (6)	-	-	-

The diazepam data were obtained from Hsu et al., 2021 [6]. The other data were generated in this study. The human serum albumin concentration sequence of each drug: ^a^ Diazepam sequence: 0%, 0.025%, 0.04%, 0.05%, 0.075%, 0.1%, 0.5%, and 2%. ^b^ Diclofenac sequence: 0%, 0.0025%, 0.01%, 0.025%, 0.1%, and 2%. ^c^ Rosuvastatin sequence: 0%, 0.1%, 0.25%, 0.375%, 0.5%, 1%, and 2%. ^d^ Fluoxetine and tolbutamide sequences: 0%, 0.025%, 0.1%, 0.25%, and 2%.

**Table 3:** Degree of fit of different models and HSA subgroups based on the sum of squares.

Drug	Interval of HSA Concentration (%)	Optimal Model	Modified WSM	WSM	PTM	DM
Diazepam	0–0.04	MWSM	0.0481	0.1123	0.0894	0.1003
	0.04–2	WSM	1.5150	0.1818	0.4375	0.2781
Diclofenac	0–0.01	MWSM	0.0278	0.0885	0.0702	0.0792
	0.025–2	WSM	0.2923	0.1194	0.1790	0.1400
Rosuvastatin	0–2	MWSM	0.2132	1.8440	1.0670	1.4550
Fluoxetine	0–2	WSM	5.9630	0.1154	0.2096	0.2062
Tolbutamide	0–2	WSM	0.0923	0.0672	0.0790	0.0715

Data for diazepam were obtained from a study by Hsu et al., 2021 [6]. The other data were generated in this study. WSM: well-stirred model; PTM: parallel-tube model; DM: dispersion model; HSA: human serum albumin.

**Table 4:** Multinomial logistic regression datasets.

Drug	MW ^a^	AlogP ^a^	PSA ^a^	Rotatable Bonds ^a^	HBA ^a^	HBD ^a^	Aromatic Rings ^a^	Heavy Atoms ^a^	V_ss_ ^b^	MRT ^b^	logCL_int,u,in-vivo_ (pred)		*fu*_p_	Class ^a^	OATP ^c^	P-gp ^c^
											hep	mic				
Diazepam	285	3.15	32.67	1	3	0	2	20	1.00	44.00	0.93	0.95	0.021	N	Without	With
Diclofenac	296	4.36	49.33	4	3	2	2	19	0.22	1.00	2.24	2.17	0.004	A	Without	Without
Rosuvastatin	482	2.4	140.9	10	9	3	2	33	1.70	2.60	1.30	NF	0.129	A	With	Without

NF: not found; S: group of compounds with similarly accurate prediction in both hepatic models; M: group of compounds with better prediction using the modified well-stirred model; W: group of compounds with better prediction using the well-stirred model; MW: molecular weight; AlogP: partition coefficient; PSA: polar surface area; HBA: hydrogen-bond acceptor; HBD: hydrogen-bond donor; V_ss_: volume of distribution at steady state; logCL_int,u,in-vivo_ (pred): logarithm of predicted in vivo unbound intrinsic clearance; hep: hepatocytes; mic: microsome; *fu_p_*: fraction unbound in plasma; A: acidic; N: neutral; OATP: organic anion-transporting polypeptide; P-gp: p-glycoprotein. ^a^ Data obtained from the EMBL-EBI database. *R_b_* was set to 1 for basic or neutral compounds and 0.55 for acidic compounds when the experimental value was unavailable in the original studies [33,34]. ^b^ Data obtained from Lombarbo et al. [35] and DrugBank. ^c^ Data obtained from El-Kattan et al. [36] and DrugBank.

## Data Availability

The authors declare that all data supporting the findings of this study are contained within the article or Supplementary Materials.

## Supplementary Materials

The following supporting information can be downloaded at: https://www.mdpi.com/article/10.3390/ph18070991/s1, Table S1: Extraction recovery (%) of diazepam, diclofenac, rosuvastatin, fluoxetine, and tolbutamide determined by LC–MS/MS analyses (n = 3); Figure S1: Calibration curves of the tested drugs (A–E) used in IPRL analysis; Figure S2: Liquid chromatography mass spectrometry of diazepam, diclofenac, rosuvastatin, fluoxetine, tolbutamide, and internal standard (IS) in Krebs buffer.

## Abbreviations

The following abbreviations are used in this manuscript:

*C_DF_*	Driving force concentrations
*C_DM_*	Representative drug concentration in DM
*C_H_*	Total drug concentration in liver
*C_in_*	Drug concentration entering liver
*CL_H_*	Hepatic clearance
*CL_int_*	Intrinsic clearance
C_MWSM_	Representative drug concentration in MWSM
*C_out_*	Drug concentration exiting liver
C_PTM_	Logarithmic mean drug concentration in PTM
C_WSM_	Representative drug concentration in WSM
DM	Dispersion model
ER	Extraction ratio
*F_H_*	Hepatic availability
*f_u_*	Intravascular fraction of unbound drug
*f_u,E_*	Extravascular fraction of unbound drug
HSA	Human serum albumin
IPRL	Isolated perfused rat liver
IVIVE	In vitro-to-in vivo extrapolation
MWSM	Modified well-stirred model
PTM	Parallel-tube model
*Q_H_*	Hepatic blood flow
WSM	Well-stirred model
