# Supplementary material for: Selection of an Optimal Metabolic Model for Accurately Predicting the Hepatic Clearance of Albumin-Binding-Sensitive Drugs
Source: Pharmaceuticals (Basel). 2025 Jul 1;18(7):991. doi: 10.3390/ph18070991 (PMC12299470; doi:10.3390/ph18070991)
Supplement: Supplementary file 1 [file pharmaceuticals-18-00991-s001.zip › pharmaceuticals-3689010-supplementary.pdf]

Supplementary Materials

Selection of an Optimal Metabolic Model for Accurately Predicting the Hepatic Clearance of Albumin-Binding-Sensitive Drugs

Ren-Jong Liang [1,2], Shu-Hao Hsu [3], Hsueh-Tien Chen [3], Wan-Han Chen [3], Han-Yu Fu [3,4], Hsin-Ying Chen [5], Hong-Jaan Wang [2,3,5,*,†], Sung-Ling Tang [2,3,4,5,*,†]

1 Clinical Pharmacy Department, Tri-Service General Hospital Keelung Branch, Taiwan, Republic of China
2 Graduate Institute of Medical Science, National Defense Medical Center, Taipei, Taiwan, Republic of China
3 School of Pharmacy, National Defense Medical Center, Taipei, Taiwan, Republic of China
4 Department of Pharmacy Practice, Tri-Service General Hospital, Taipei, Taiwan, Republic of China
5 Graduate Institute of Life Science, National Defense Medical Center, Taipei, Taiwan, Republic of China
* Correspondence: hongjaan@mail.ndmctsgh.edu.tw; Tel.: +886-2-8792-3100 (ext. 18849)
* Correspondence: tangling@mail.ndmctsgh.edu.tw; Tel.: +886-2-8792-3100 (ext. 18856)
† These two authors contributed equally to this work.

List of content:

Table S1. Extraction recovery (%) of diazepam, diclofenac, rosuvastatin, fluoxetine, and tolbutamide determined by LC–MS/MS analyses (n = 3).

Figure S1. Calibration curves of the tested drugs (A–E) used in IPRL analysis.

Figure S2. Liquid chromatography mass spectrometry of diazepam, diclofenac, rosuvastatin, fluoxetine, tolbutamide, and internal standard (IS) in Krebs buffer.

Table S1. Extraction recovery (%) of diazepam, diclofenac, rosuvastatin, fluoxetine, and tolbutamide determined by LC–MS/MS analyses (n = 3)

Analyte	Concentration (ng/mL)	Recovery (%)	CV (%)
**Diazepam**	1000	80.1±2.4	3.01
**Diclofenac**	1000	50.7±1.7	3.44
**Rosuvastatin**	1000	68.8±0.9	1.27
**Fluoxetine**	1000	63.0±4.3	6.83
**Tolbutamide**	1000	81.7±0.8	0.95

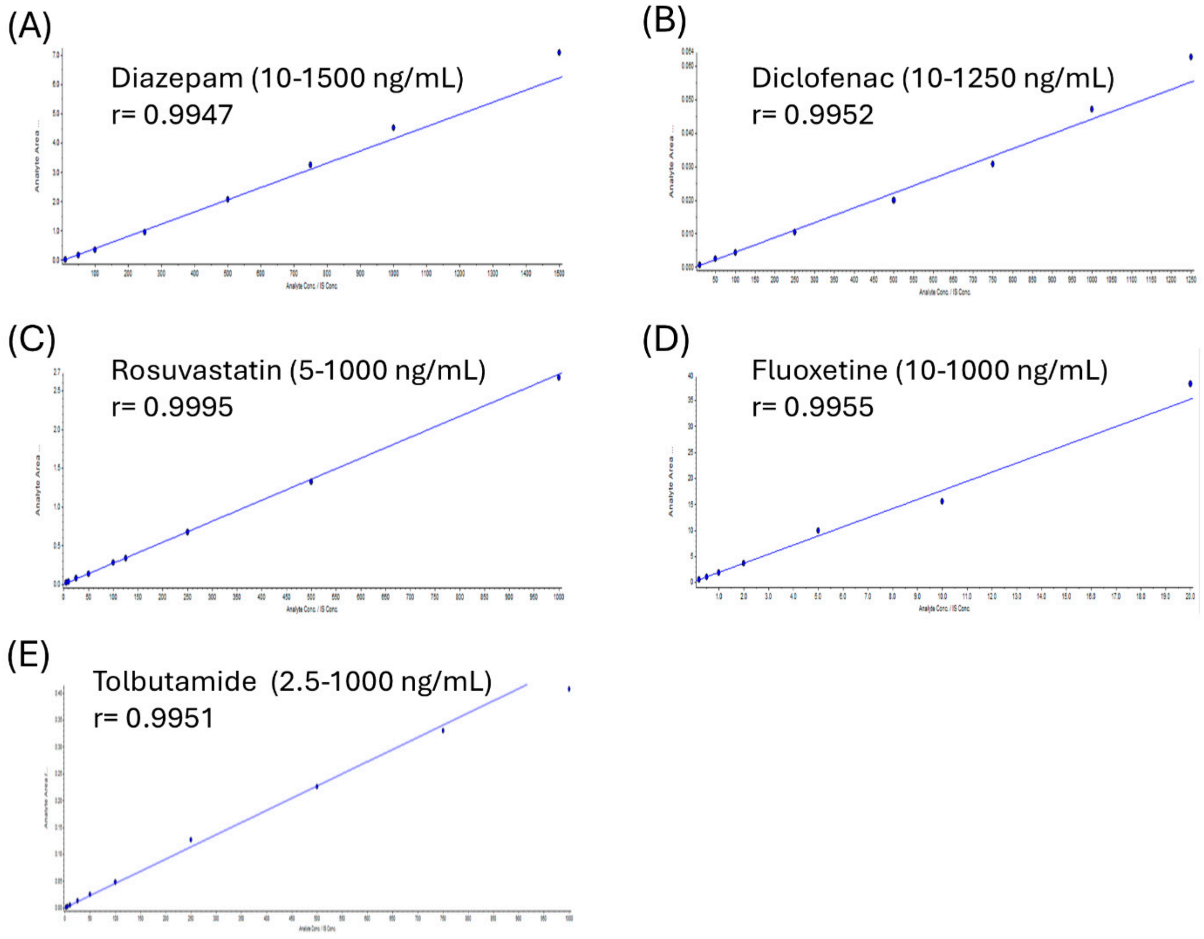

Figure S1. Calibration curves of the tested drugs (A–E) used in IPRL analysis. Calibration ranges are indicated in parentheses, and correlation coefficients (r) are provided to demonstrate the linearity of each curve.

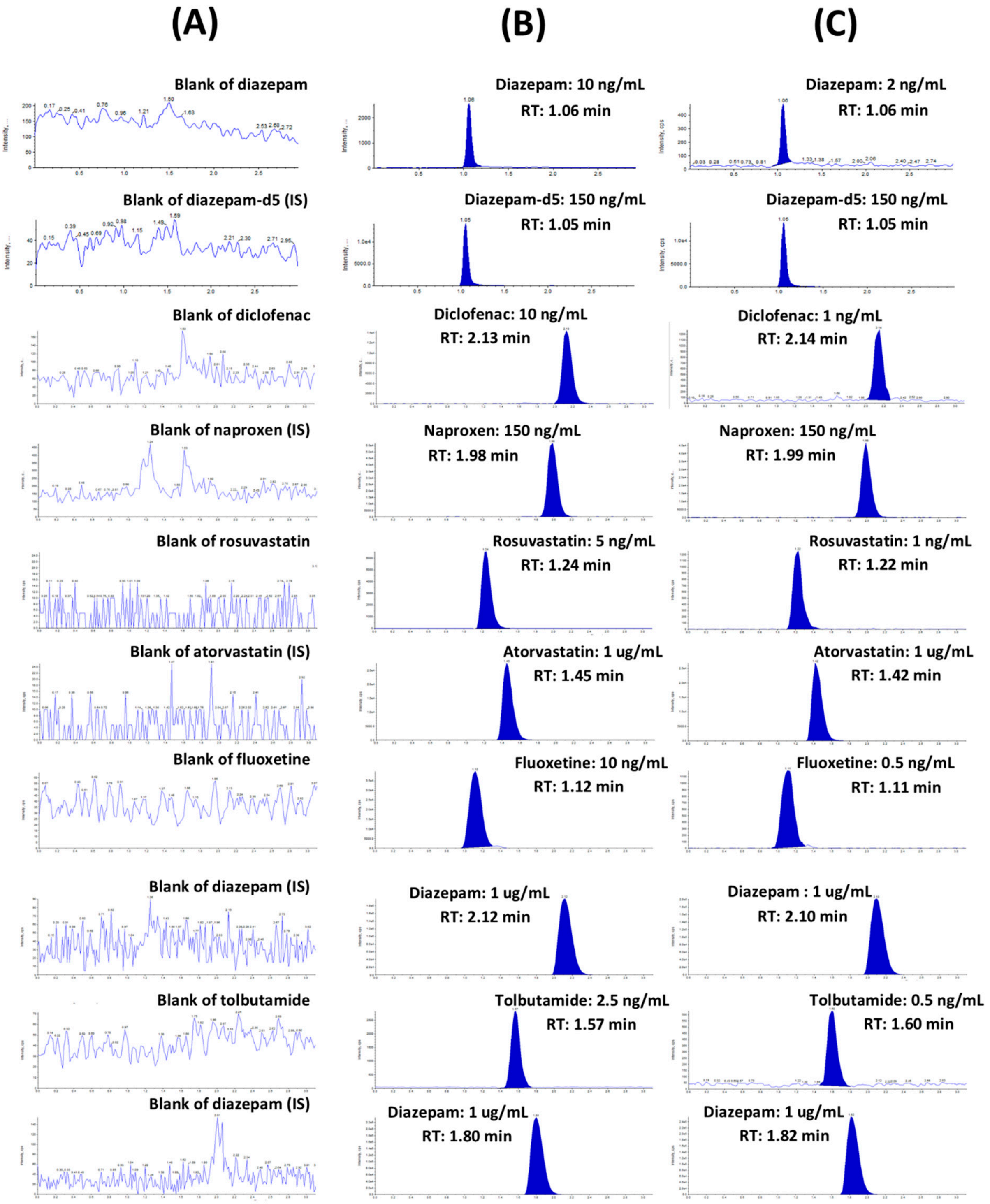

Figure S2. Liquid chromatography mass spectrometry of diazepam, diclofenac, rosuvastatin, fluoxetine, tolbutamide, and internal standard (IS) in Krebs buffer. MRM chromatograms of (A) blank Krebs buffer; (B) blank Krebs buffer spiked with analytes and IS at LOQ; (C) blank Krebs buffer spiked with analytes and IS at LOD.
